# Impact of Physical and Social Frailty on the Utilization of Nursing Care Services in Very Old Adults

**DOI:** 10.1093/geroni/igab046.2193

**Published:** 2021-12-17

**Authors:** Jaroslava Zimmermann

**Affiliations:** University of Cologne, Cologne, Nordrhein-Westfalen, Germany

## Abstract

Frailty, characterized by increased vulnerability to external stressors, has been found to increase the risk of healthcare utilization and nursing home admission. As the age group of 80 years or older remains frequently underrepresented in previous research, this study examined the impact of physical and social frailty on the utilization of nursing care services in very old population of North Rhine-Westphalia. Using data from a representative cross-sectional study, 1,577 community-dwelling and institutionalized individuals aged ≥80 years were included. Physical frailty was defined according to Fried’s criteria (exhaustion, weight loss, low handgrip strength, low physical activity). Social frailty was measured with self-reported loneliness, social isolation, and time spent with others. The use of outpatient care services, day care, informal and inpatient care were considered. Multinomial regression was applied to investigate the impact of physical and social frailty on the use of outpatient and inpatient care services, controlling for relevant sociodemographic and health related characteristics. Compared to very old adults who did not use any care services, no association was found between frailty and the use of outpatient or informal care. Comparing nonusers of care services with institutionalized individuals, nursing home residents were less likely to experience physical frailty and pre-frailty, but were more likely to be socially isolated and to feel lonely. These findings suggest that physical frailty might have been successfully prevented in the context of institutional inpatient care. However, early identification and intervention focused on social inclusion of the institutionalized very old individuals are needed to reverse social frailty.

